# Meeting the meat: delineating the molecular machinery of muscle development

**DOI:** 10.1186/s40781-016-0100-x

**Published:** 2016-05-10

**Authors:** Arif Tasleem Jan, Eun Ju Lee, Sarafraz Ahmad, Inho Choi

**Affiliations:** School of Biotechnology, Yeungnam University, Gyeongsan, 712-749 Republic of Korea

**Keywords:** Muscle, Muscle satellite cells, Muscle differentiation, Trans-differentiation

## Abstract

Muscle, studied mostly with respect to meat production, represents one of the largest protein reservoirs of the body. As gene expression profiling holds credibility to deal with the increasing demand of food from animal sources, excessive loss due to myopathies and other muscular dystrophies was found detrimental as it aggravates diseases that result in increased morbidity and mortality. Holding key point towards improving the developmental program of muscle in meat producing animals, elucidating the underlying mechanisms of the associated pathways in livestock animals is believed to open up new avenues towards enhancing the lean tissue deposition. To this end, identification of vital candidate genes having no known function in myogenesis, is believed to increase the current understanding of the physiological processes going on in the skeletal muscle tissue. Taking consequences of gene expression changes into account, knowledge of the pathways associated with their activation and as such up-regulation seems critical for the overall muscle homeostasis. Having important implications on livestock production, a thorough understanding of postnatal muscle development seems a timely step to fulfil the growing need of ever increasing populations of the world.

## Background

Nutrition significantly influences the physical endurance and performance of living beings. For maximum performance, a diet that can provide a minimum half of our daily energy intake in the form of proteins and carbohydrates is recommended. Differences in food choice for optimal nutritional balance have led to selection of substrates of animal origin rich in proteins. To meet the modest increased interest in food from animal sources, most of the studies in livestock remain confined for being the main contributor of meat. Therefore, maintaining a balance between supply and demand requires a good understanding of muscle regulation to enable improved muscle mass without compromising animal health and meat quality. As contribution of livestock to mankind goes beyond food production to multipurpose uses, it became necessary to have a deep insight of the genetic machinery that regulates diverse cellular functions in consideration with their important economical role [1]. In this regard, studies pertaining to muscle development using a wide range of experimental models systems have opened up new avenues to gain better insight into changes in gene expression during different stages of growth and development.

Technological advancement that has led to methods such as microarrays, have shifted studies more toward global gene expression profiling ([2, 3] and References therein). Rather than utilizing the C2C12 cell line, primary cells compatible with in vivo environments were used to get accurate information regarding the roles of genes in muscle development [3]. Knowing the fact that factors responsible for muscle depot traits have pronounced effects on the taste and palatability of meat, a study was directed to investigate differences in the expression patterns of proteins among bovine primary muscle satellite cells (MSCs) from beef shank (BS), *longissimus dorsi* (LD), *deep pectoral* (DP) and *semitendinosus* (ST) muscle of Hanwoo cattle [2]. Microarray, expressed sequence tagging (EST) and RNA sequencing revealed information about importance of certain novel genes with unknown function, especially with regard to their differential expression in the myogenic program. Using primary cell culture system, gender-specific effect of serum components on the proliferation and differentiation of MSCs revealed male serum (MS) enhancing the proliferation and differentiation of MSCs, while female serum (FS) enhancing lipid accumulation associated with the taste and palatability of adipocyte-like cells (ALCs) [4–7]. Taken together, importance of differentially expressed genes such as fibromodulin (FMOD), matrix Gla protein (MGP), and transthyretin (TTR) with respect to regulation of the myogenic program was reported [2, 8–10]. Identification of differentially expressed genes eventually led to increased interest in studying their roles in the differentiation and trans-differentiation processes. Although studies establishing their roles in muscle development are still ongoing, studies are currently being pursued to delineate the accurate mechanism through which they act [11, 12]. The present study was aimed to bring out state of art information regarding satellite cells and of the genes that play important role with respect to regulation of muscle cell program.

## Satellite cells in muscle development

Muscle in all its forms makes up nearly half the body’s mass. It is endowed with features of excitability (active response to stimulus), contractility (contraction upon stimulus), extensibility (stretchable capacity) and elasticity (ability to recoil and regain its normal length) that enable it to function [13]. In an intact muscle, the individual muscle fibres are wrapped and held together by connective tissue sheaths; namely, the *epimysium*, which is the outermost layer surrounding the entire muscle, the *perimysium* covering individual bundles or fascicles inside the *perimysium,* and the *endomysium*, which surrounds individual muscle fibres within fascicles. Together, these connective tissue sheaths support each cell and reinforce the muscle as a whole. Among the three muscle types, skeletal muscle represent highly adaptive tissue endowed with the ability to alter muscle mass and fiber size via the addition of new myonuclei in response to physiological stimuli. Being a form of striated muscle, it contributes to function and dysfunction of the musculoskeletal system. Although activated by reflexes, skeletal muscles composed of multinucleated myofibers are subjected to conscious (voluntary) control. Their fitness is correlated with two healthy states, strength and muscular endurance. Strength represents the force capacity and muscular endurance the ability to contract without getting exhausted.

Muscle cells, which are believed to be remnant embryonic myoblasts, comprise a population of muscle-specific progenitors that possess extraordinary regenerative capacity. Technological advancement has led to track their original location across a broad range of vertebrate species including mice [14–16], chicken [17, 18], rat [19] and humans [20, 21]. Addition to generation of purified populations of MSCs using advanced techniques such as fluorescent-activated cell sorting (FACS), expression of β-galactosidase (β-gal) or fluorophores (e.g., GFP) using nestin regulatory circuits has made their monitoring possible, even in freshly isolated myofibers [22, 23]. Studies investigating the fusion of mononucleated myoblasts for generation of multi-nucleated myofibers set the stage for the current understanding of regeneration [24, 25]. Subsequent studies conducted by Konigsberg et al., [26] and Bischoff [27] provided substantial evidence of myofiber harbouring cells having the potential to give rise to myoblasts and multi-nucleated myotubes. Confirmation of sharing a similar anatomic position across the majority of vertebrates has led to acceptance of its candidature as source of myogenic cells necessary for postnatal growth.

**Fig. 1:**
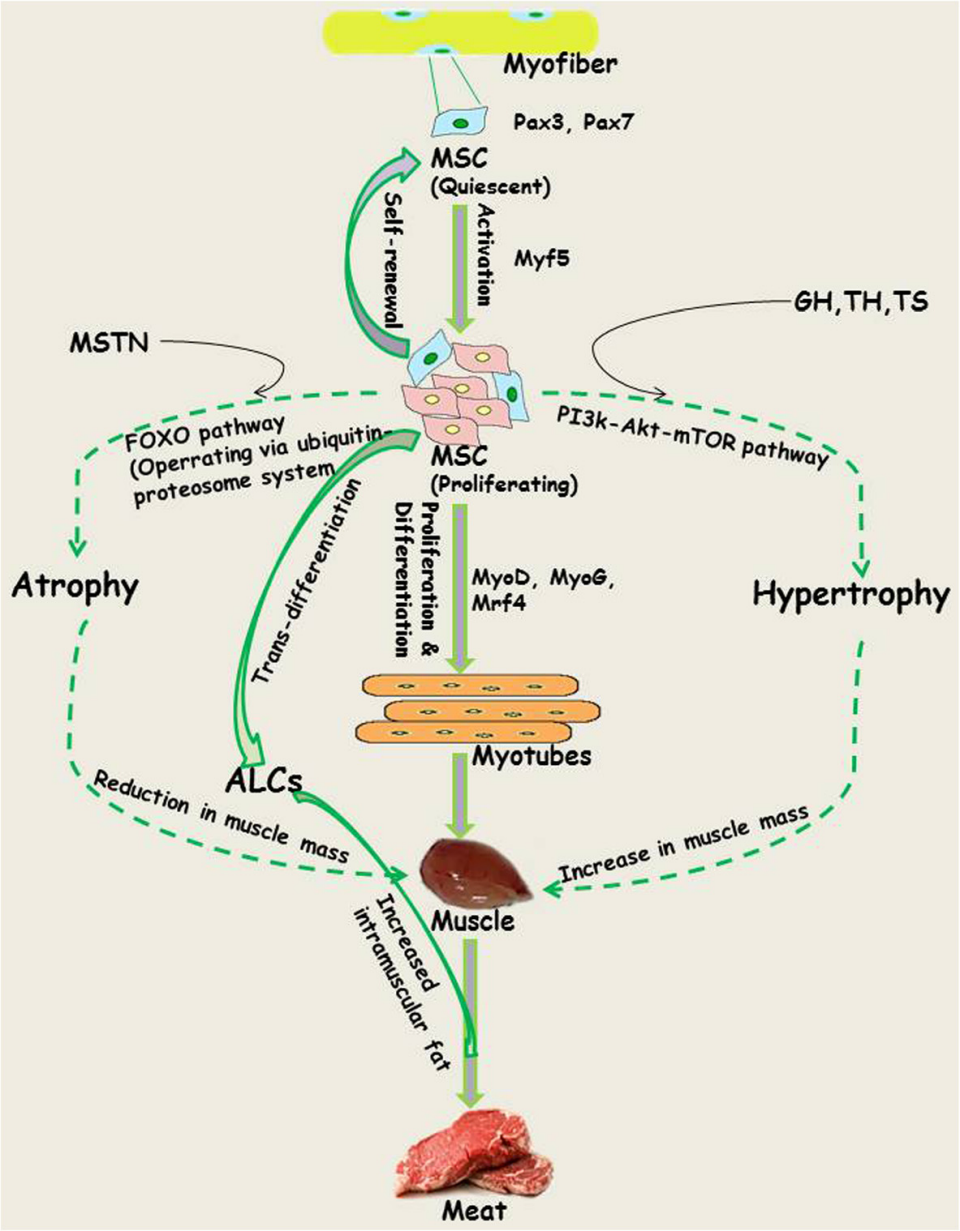
A story of 3 M’s (MSC’s, Muscle and Meat). An outline depicting phases of transition of MSCs along with the role of transcriptional factors, genes and growth enhancing substances (hormones) associated the myogenic program

Skeletal muscle originates from the mesodermal cells of somites and its development begins with the commitment of muscle satellite cells (MSCs) surrounding each myofiber to proliferate and differentiate to myoblasts. Despite the fact that myofiber number remains constant during early (neonatal/juvenile) stages of life, their contribution to growth is attributed to fusion of MSCs. MSCs represent 30 % of the nuclei during early postnatal growth [17, 28]. Being the main contributor to immediacy and sensitivity of skeletal muscle, MSCs play a key role in maintenance of the structural and functional integrity of muscle (Fig. 1). Subsequent to activation, about 80 % (responsive population) of the MSCs enter the cell cycle phase, while the remaining 20 % (reserve population) that represent the true stem cell population undergo symmetric divisions to replenish the quiescent cell pool [29, 30]. Progression of MSCs along the myogenic lineage commences with the co-expression of paired box transcription factors, Pax3/Pax7, followed by contribution from basic helix loop helix (bHLP) family of transcription factors, commonly referred as myogenic-regulatory factors (MRFs; including Mrf4, Myf5, MyoD and myogenin; MyoG) [31, 32]. The fate of MSCs is determined by changes in the pattern of expression of MRFs. For example, down regulation of Pax7 and upregulation of Myf5/MyoD followed by MyoG commits them to the myogenic program, while absence of MRFs leads to retention of the quiescence state among the satellite cell population [32, 33]. Regardless of origin, MSCs share Pax7 expression across all muscles [34, 35]. Although exclusively expressed in the subpopulations of quiescent MSCs, the role of Pax3 is restricted to proliferation via induction of Myf5 because it does not play any indispensable role in quiescence. The stochastic down regulation of MyoD to retain the state of quiescence under in vitro conditions is interpreted as the underlying mechanism for self-renewal of MSCs. Overall, it is the network of transcriptional factors that appears to control the progression of MSC lineage from origin towards myogenic specification, differentiation and then fusion to generate myoblasts.

## Myogenesis in livestock

Subjected to intensive selection for improvement in terms of quality and quantity of meat, livestock breeds (cattle, sheep, poultry) represent useful genetic model for the study of hypertrophy and other muscle traits [36, 37]. Although the extent of muscle cell multiplication determines how many muscle fibres are formed, the fate of growing muscle fibres during myogenesis depends largely on the extent of progenitor muscle cell multiplication. Being dependent on the number and proliferative stage of progenitor cells, maternal nutrition is known to have a dramatic effect on the development of skeletal muscle during this stage [38–41]. Prenatal myogenesis is divided into primary (embroyonic; during which primary muscle fibres arise) and secondary (fetal; leading to secondary muscle fibres) myogenesis. However, only a small fraction of primary muscle serves as template for secondary muscle fibres. Growth selection that led to increases in the proliferation rates of myoblast and/or satellite is represented by increased myonuclear numbers and higher muscle DNA content. Muscle fibres formed during the prenatal stage contributes significantly to growth and development of muscle in livestock. As such, genetic and environmental factors capable of influencing prenatal myogenesis act as a determining factor to the number of muscle fibres in any muscle. Muscle fibres number being essentially fixed at birth; therefore, postnatal growth results from the hypertrophy of existing muscle fibres in meat-producing animals [42, 43]. For contribution to hypertrophy of muscle fibres, nuclei being unable to divide are procured for incorporated into muscle fibres through the proliferative activity of MSCs. Hence, understanding the mechanism pertaining to prenatal development of skeletal muscle is important as it is well known for its dramatic impact on postnatal growth and development.

Increased muscle fibre number, which is attributed to the proliferative activity of MSCs during prenatal growth, markedly determines the growth and development capacity of postnatal muscles. However, increase in the mass during postnatal growth occurs due to increased muscle fibre size (length and girth) rather than muscle fibre number. Accordingly, MSCs remain the sole source that contributes for increase in the muscle fiber size. In addition to the increase in muscle fiber size, fusion of MSCs generates myoblasts with pre-existing muscle fibres that contribute to an increased number of nuclei in adult muscle fibres. Postnatal growth rate is inversely correlated with muscle fibre number, as muscle development was found lower due to high fibre numbers and higher owing to low number of fibres. It has been suggested that this occurs owing to reduced energy and oxygen supply by lower capillary density, as well as impairment of the nuclear control of cellular processes due to low nuclear: cytoplasm ratio [44–46]. In short, there is clear antagonism between muscle fibre number and thickness, with low fibre number correlating with fibres that exhibit higher hypertrophy. It has been reported that muscle hypertrophy that lead to increase in muscle mass reduces the stress adaptability of fibres, which in turn is associated with poor meat quality of major livestock breeds [46–49]. Hence, having a better knowledge of the involvement of MSCs in postnatal hypertrophy is essential to improve the efficiency of muscle growth in meat-producing animals.

## Muscle homeostasis: balance of atrophy and hypertrophy

Muscles are highly adaptable owing to their remarkable plasticity. They arise as multinucleate syncytium from the fusion of mononuclear myoblasts. An intricate balance between production and degradation of myofibrils is crucial to muscle growth and maintenance of healthy state [50, 51]. In addition to mammals, studies of Drosophila highlighted the role pertaining to balance in the protein content (synthesis and breakdown) in determining the fate of muscle tissue [51]. By regulating the number of cells capable of undergoing proliferation, MSCs modulate myofiber growth accordingly. To ensure constant and harmonious growth of all muscles, cellular turnover (addition of new myonuclei via fusion of satellite cells) in addition to maintenance of proteins appears crucial during early embryonic development in the muscle development program. Contribution of cellular turnover to the homeostasis of adult fibres is achieved through increased synthesis rather than degradation of proteins. Adhering to this, the interrelated processes are tightly regulated 1) at the level of protein synthesis through degradation of the misfolded proteins, and 2) at the level of protein degradative machinery required to replace proteins as a result of changes in muscle activity. The net difference in these two processes contributes to gross protein deposition, which is manifested as muscle hypertrophy.

Coordinated regulation in the pathways controlling synthesis and degradation of muscle proteins strongly influences the physical endurance of the growing muscle [52]. In efforts to elucidate the pathways controlling cellular and protein turnover, application of genetic approaches for gain or loss of function mutation has helped in establishing their role in muscle hypertrophy or reduced muscle growth [51]. Muscle specific over-expression of insulin like growth factor 1 (IGF-1) or Akt by employing a transgenic module through electroporation leads to restoration of muscle growth with matching physiological strength and displays higher regeneration potential for sustainable muscle growth [53, 54]. IGF-1 is known to activate mitogen-activated protein kinase/extracellular signal reduced kinase (MEPK/ERK) and phosphoinositide 3-kinase (PI3)-Akt pathways, which are in turn associated with the induction of muscle growth either by stimulating protein synthesis through mammalian target of Rapamycin (mTOR) or by inhibiting the degradative machinery induced by forkhead box O (FOXO) transcription factors. Upon activation by Akt, mTOR act through two different branches of the Akt pathway; Rapamycin-sensitive TORC1 containing raptor and Rapamycin-insensitive TORC2 containing rictor ([55] and references therein). Conversely, reduced PI3k signalling causes decreased protein synthesis machinery and a subsequent increase in proteolysis through FOXO mediated expression of the atrogene program. In addition to reduced PI3k signalling during fasting and in diseased state, increased expression of myostatin (MSTN) leads to inhibition of PI3k-Akt signalling [54]. Overall, this pathway has a positive effect on regulation of muscle growth by stimulating protein synthesis and inhibiting the degradation trigger of proteins by the FOXO system via the ubiquitin-proteosome system (UPS) and autophagy (Fig. 1).

MSTN (also referred as growth and differentiation factor-8, GDF-8), a member of the transforming growth factor-β (TGF- β) family expressed and secreted by skeletal muscle tissues, is an endogenous negative regulatory factor that regulates growth and development of the muscle [56–58]. Its function as a negative regulator was first reported by McPherron et al., [59] through studies on MSTN null (MSTN^-/-^) mice, exhibiting double mass attributed by combination of increased number (hyperplasia) and size (hypertrophy) of muscle fibres. This effect was observed through regulation of muscle fiber sizes rather than contribution from MSCs, which are devoid of MSTN receptors [60–62]. MSTN, which is expressed primarily by myotubes, acts as an inactive precursor protein in the extracellular matrix of muscle and/or remains in circulation as an endocrine hormone until it undergoes proteolytic cleavage followed by dimerization of the *C*-terminal to generate active MSTN [63, 64]. However, until its release from the cell it remains inactive through the formation of a latent complex with the *N*-terminal pro-peptide [65–67].

Subsequent to activation (removal of *N*-terminal pro-peptide), mature myostatin binds to its transmembrane receptor, activin receptor protein type IIB/A (ActRIIB/A), which undergoes dimerization with activin type I receptors (ALK-4/5) to induce signalling in the internal cellular environment through Smad proteins [68–70]. Activation of Smad2 and Smad3 lead to heterodimer complex formation with the common mediator, Smad4. Subsequent to translocation to the nucleus, Smad complex (Smad2,-3,-4) activates transcription of target genes though interaction with the DNA [71, 72] or block the fusion of proliferating myoblasts after transactivation of MyoD [73, 74]. Additionally, Smad complex activates signalling along the Erk1/2 MAPK pathway to prevent myoblasts proliferation via p21/Rb signalling cascade and promotion of anti-apoptotic pathways via activation of p53 in differentiated cells. Upon activation, Smad7 (inhibitory Smad protein) functions as a negative feedback inhibitor for the MSTN signalling pathway [58, 75, 76].

Corresponding to muscle progenitor cells, MSTN maintains a fine balance between proliferation and differentiation; however, any disturbance to this balance changes this to either differentiation (in the case of over-expression) or proliferation (inhibition of MSTN), which leads to expansion of progenitor cell pool. Furthermore, reports have suggested that inhibition of the PI3K/Akt/mTOR signalling pathway occurs via phosphorylated SMAD3 through induction of the E3-ligase, atrogin-1 [77, 78]. Another study reported that induction of the degradation of muscle protein through ubiquitin-proteasome machinery inhibit the PI3K/Akt/mTOR signalling pathway [79]. Taken together, these studies suggest the possibility of cross-talk at different levels between MSTN and PI3K/Akt/mTOR signalling pathways.

## Hormonal regulation of muscle mass

Regulation of growth occurs through the substantial involvement of neuro-endocrine system as well as local autocrine/paracrine actions of hormones and growth factors. These compounds elicit their effects either through changes in their local production or with respect to their activity in controlling the myogenic stages of muscle cells. A key prerequisite for optimised muscle growth involves the balanced secretion of two hormones, growth hormone (GH) and testosterone (T). Growth hormone (GH) stimulates growth, cell reproduction and regeneration in humans and other animals. Used mainly as a performance enhancing drug, GH stimulates production of IGF-1, a peptide hormone homologous to proinsulin from the liver through JAK-STAT pathway ([80] and references therein). Acting via IGF-1, GH produces its effect through the stimulation of MSC proliferation along with synthesis of proteins in muscle. The growth stimulating effects of IGF-1 have been reported from a wide variety of tissues. Postnatal application of GH leads to an increase in lean growth and decrease in fat deposition, and often stimulates muscle fibre hypertrophy [81, 82].

To accomplish this, the most frequent mechanism of change in thyroid hormone (TH) level is GH-mediated increase in T_4_ to T_3_ conversion by means of deiodination in target tissues [10, 83]. Binding of THs to their distributor proteins (thyroxine-binding globulin (TBG), transthyretin (TTR) and albumin) facilitates their distribution to a greater extent. TBG exhibiting highest affinity (1.0 × 10^10^ M^−1^ and 4.6 × 10^8^ M^−1^) leads the group for transporting T4 and T3, followed by TTR (7.0 × 10^7^ M^−1^ and 1.4 × 10^7^ M^−1^) and then albumin (7.0 × 10^5^ M^−1^ and 1.0 × 10^5^ M^−1^) [84]. Taken together, all three proteins constitute a buffering network for T4 in the blood, which provides a means of protection against fluctuation in the level of THs that can led either to hypothyroidism or hyperthyroidism. Regardless of variation in the concentration of distributing proteins, TBG (0.015 g/l) is known to distribute major proportion (up to 75 %) of T4 and T3 followed by TTR (0.25 g/l) and albumin (42 g/l), which distribute 15 % and 10 %, respectively (Alshehri et al. [84]). Concentration of TH distributor proteins having high binding affinity for THs prevents partitioning of THs into the lipid component of the cell membrane, thereby ensures a continuous pool of circulating THs in the blood [85, 86].

Although synthesis of TTR is restricted to the liver, its synthesis has also been reported in the choroid plexus of brain and in muscle tissues [2, 9, 87, 88]. Besides transporting thyroxine, TTR is known to assist in the transport of retinol through its binding to retinol-binding protein (RBP) [89]. Having dual role in transport thyroxine and retinol fetches it with the name TransThyRetin. In mammals, TTR possesses higher binding affinity for T4 than its active form, T3, while all other vertebrate species exhibit higher binding affinity of TTR for T3 than T4. Transit times for delivery of THs to tissues vary according to the binding affinities of different TH distributor proteins. As such TTR having intermediate affinity is responsible for immediate delivery of THs to tissues compared to TBG, which holds THs tightly and albumin, which binds THs loosely to deliver them at their specific sites [9, 84]. At the tissue level, particularly in muscle, TTR was found to play a critical role in transporting and delivering T4 to cells. Using knockdown approach, we demonstrated that TTR was more involved in differentiation than proliferation. During differentiation, TTR was found to be associated with regulation of the expression of early stage genes such as myosin light chain 2 (MYL2), as well as in affecting the functioning of Ca^2+^ channel genes such as Cav1.1 and Cav3.1 [8, 10].

TTR is highly conserved in terms of structure across a broad range of species. Once reaching the respective destination, it is acted upon by a family of deiodinases that either activates it via T4 to T3 conversion or inactivates it following conversion to either T2 or rT3 [90]. Following binding to thyroid receptors (TRs), T3 undergoes translocation to the nucleus, where it acts on the promoter region of certain specific genes, thereby causing dissociation of corepressor molecules or association of coactivator proteins. Secretion of T4 from thyroid gland contributes to first level T3 availability to cells. The second level of regulation occurs at the peripheral level, where TH transporters (MCT8, MCT10 and OATP1C1) and TH metabolising enzymes (deiodinases) regulate intracellular T3 levels in a tissue-specific fashion.

The growth promoting effects of steroids (anabolic hormones) are well known in cattle, whereas limited effects are known from swine [91]. Indeed, steroids have long been used to improve the efficiency and product quality of animal meat. Testosterone (TS) is known for its ability to control the number and size of muscle fibre. TS has also been found to stimulate growth of existing myofibres longitudinally to increase number of fibre per cross sectional area of muscle. Postnatal application of testosterone stimulates muscle fibre hypertrophy directly or indirectly without increasing fibre number through stimulation in the satellite cell proliferation and synthesis of muscle proteins [4, 92–94]. However, in the case of males and females, difference in fibre number arises via hormonal action during prenatal period of fibre formation. While the male sex hormone plays an important role in enhancing proliferation and differentiation of MSC, the female sex hormone is influential in the transition of MSCs towards transdifferentiation, which causes lipid accumulation in differentiating myotubes [94, 95]. Increased intramuscular fat is desirable in the meat industry as it marginally increases tenderness, juiciness and flavor intensity. Based on this knowledge, growth-promoting agents that can influence muscle growth in farm animals have been derived. There have also been reports of growth-promoting effects exerted by β-adrenergic agonists among various species [96–99]. In contrast to GH, most β-adrenergic agonists are not able to stimulate MSC proliferation, and instead produce short term effects by increasing protein synthesis in the muscle fibres.

## Meat quality: a parameter needed for consumer satisfaction

Optimal meat quality represents a complex of intrinsic (e.g. color stability, tenderness, palatability and water-holding capacity) and extrinsic (e.g. price, brand name, origin, packaging, labelling, etc) characteristics, that are meant to satisfy consumers. Optimal meat quality characteristics vary with human culture and time, with a general trend of increasing contribution to safety and healthiness that are important for consumption and economic reasons. With increasing demand for products with enhanced safety and healthiness, food production systems with improved quality standards are important to deliver and guarantee the safety of the products to consumers. Variation in meat quality arises through differences in the metabolic processes occurring in the muscles during peri- and post-slaughter period. Accordingly, muscle fibre type and capillarity within animals are important factors [46, 100]. Meat quality is generally accessed in terms of tenderness, taste, etc. Tenderness of meat is positively related to the muscle fibre characteristics (cross-sectional areas of the fibres, fibre types, metabolic enzyme activities, collagen quantity) and the amount of intramuscular fat that contribute to determination of flavor [101, 102]. Color of meat is determined by the level of myoglobin oxygenation [100]. Meat with high percentages of oxidative fibres (greater myoglobin content) has a red color. These fibres show a high level of post-mortem shortening and generally have a low glycogen content. Capillarization is another relevant factor that influences meat quality since it is associated with supply of oxygen to muscles fibres. Through this, capillarization not only influences the metabolic state at time of slaughter but also during post-slaughter period.

**Fig. 2:**
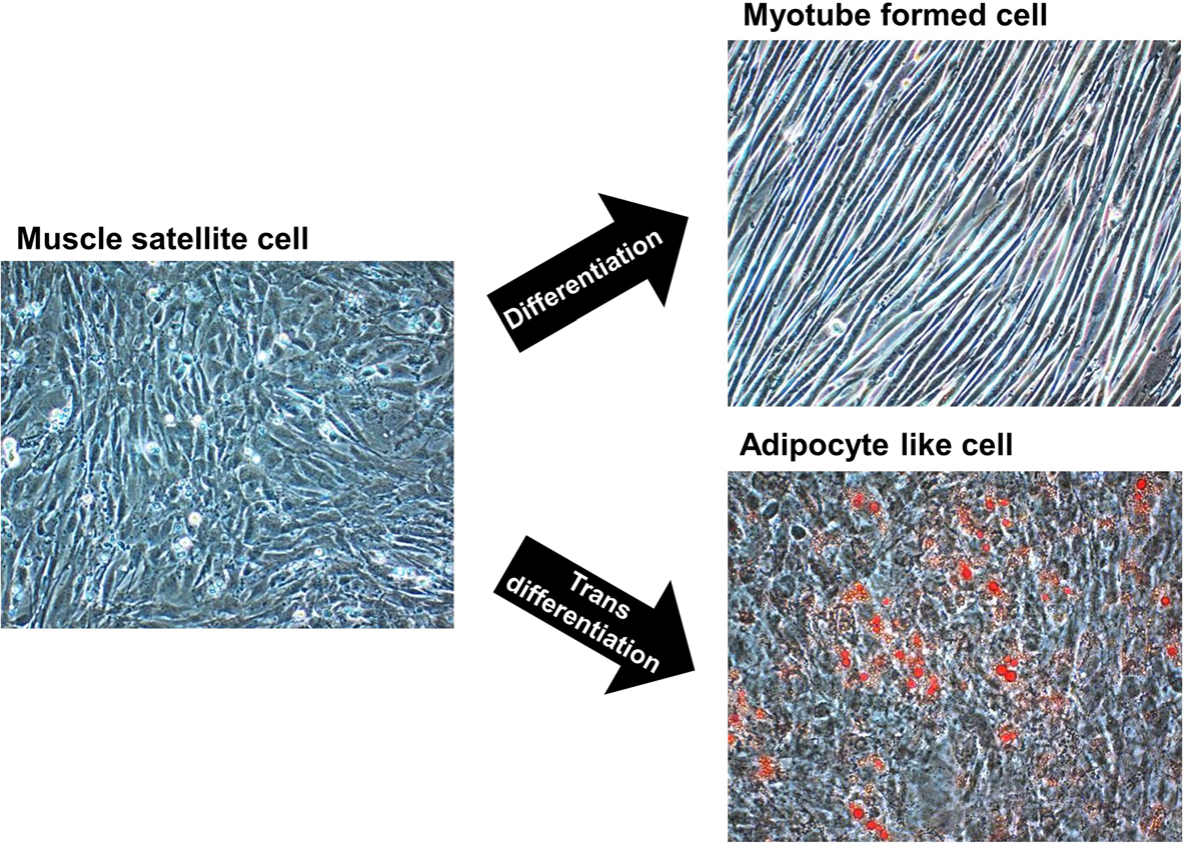
Switching of primary bovine MSC’s to myogenic (differentiation) or adipogenic (trans-differentiation) program. Left panel represent proliferating MSCs while as panel on the right indicate cells under differentiating and trans-differentiating conditions

Adipogenesis overlaps myogenesis during mid-gestation period in ruminant animals and humans [103–105]. Commitment of MSCs to adipogenic lineages (transdifferentiation) being in competition with the regular myogenic (differentiation) program, is shaped through the involvement of numerous inductive regulators (Fig. 2). Progressive loss of muscle mass that exerts negative effect on the structural and functional integrity of muscle fibre results in the decline of muscle strength [106, 107]. However, switching the commitment of MSCs from myogenic to adipogenic program during fetal muscle development results in increased intramuscular fat and therefore marbling in the offspring [40, 108–110]. Being crucial to the flavor of meat, enhancement of intramuscular fat through increased number and size of intramuscular adipocytes improves meat quality [103, 111, 112]. Growing demand for highly marbled meat has resulted in exploration of the inducers that can cause transition of MSCs towards adipose-like cells (ALCs) within muscle [3, 113, 114]. In search for inducers, role of adipogenic transcriptional factors CCAAT-enhancer binding protein alpha (*C/EBPα*) and peroxisome proliferator-activated receptor gamma (*PPARγ*) was found conserved [5, 115–117]. Considering the importance of fat in muscle to meat industry, ligands such as thiazolidinediones, which activates the function of transcription factors C/EPBα and PPARγ, are being used to induce transdifferentiation in MSCs [117–119]. While maternal over-nutrition has been found to increase adipocytes during late gestation in the skeletal muscles of fetal sheep [41, 120], controlled concentrations of serum lipids have been shown to induce transdifferentiation of MSCs into adipoblasts [5, 119].

## Conclusion

Growth rate corresponding to muscle is considered an important performance factors for evaluating profits. Studies of MSC populations have enhanced the current understanding of the regulatory mechanisms that direct somatic stem cell populations for their role in the developmental program. Advancement in the high-throughput sequencing and system biology that advanced the search for such genes have made it possible to elucidate a core regulatory network of myogenic genes that drives the myogenic fate of pluripotent stem cells. This program, though orchestrated by key transcription factors, dictates the balance between proliferation and differentiation and drives the functional transformation from individual proliferating myogenic cells to a syncytial contractile myofiber. Additionally, increasing knowledge obtained through *in silico* studies of differentially expressed genes has provided an outstanding tool for investigating the interacting network operating between different genes to elucidate the molecular machinery behind activation, proliferation and differentiation of MSCs. To meet the growing demand of the increasing population, exploiting the regulatory circuits through the use of primary culture system is believed to provide deep insight into the measures that can be employed for improving the postnatal skeletal muscle growth among different animal species. As such, understanding genetic and epigenetic regulation of cell pluripotency, reprogramming and cell differentiation/dedifferentiation seems critical to improve livestock production.
